# 

**Published:** 1905-10-21

**Authors:** 


### NOTICE TO CORRESPONDENTS.

All MSS., Letters, Books for Review, and other matters intended for the Editor should be addressed The Editor, "The Hospital," The Hospital Buildings, 28 & 29 Southampton Street, Strand, W.C.

The Editor cannot undertake to return rejected MS., even when accompanied by stamped directed envelope.

**Notice to Advertisers and Subscribers.**

All Advertisements, Orders for Copies of The Hospital, and Business Communications should be addressed to **THE MANAGER (Not to the Editor),** The Hospital, 28 & 29 Southampton Street, Strand, London, W.C.

The Cost of Subscription, post free, is at follows:—Per week, 3½d.; per quarter, 3s. 3d.; per half-year, 6s. 6d.; per annum, 13s. Foreign and Colonial:—Per annum, 19s. 6d., payable, in all cases, in advance.

**NOTICE.—Vol. XXXVII. of "The Hospital" October 1904 to March 1905), in handsome cloth binding, will shortly be on sale at the office of this Journal, price 10s. 6d. Cases for binding the Numbers contained in this Volume 2s. Index to Vol. XXXVII., 3½d., post free.**

**The Monthly Part for October is now ready. Price 1s., by post, 1s. 3d.**

### BOOKS RECEIVED.

W. B. Saunders and Co.

"Dietetics for Nurses." By J. Friendenwald, M.D., and J. Ruhräh, M.D.

Scientific Press, Limited.

"Lectures on Midwifery." By E. Stanley Hoare, M.R.C.S., D.P.H.Lond.

Wells Gardner, Darton and Co.

"A Healthy Home, and How to Keep It." By Florence Stacpoole.

Longmans and Co.

"Gray's Anatomy." 16th edition.

University Tutorial Press.

"Matriculation Directory." XLI., September, 1905

### Medical Appointments.

**Henry Brunton Angus,** M.B., M.S.Durh., F.R.C.S.Eng., Honorary Surgeon to the Royal Infirmary, Newcastle-upon-Tyne. **R. W. Brimacombe,** M.D.Brux., Pathologist to St. John's Hospital for Diseases of the Skin, Leicester Square, W.C. **G. W. K. Crosland,** M.R.C.S., L.R.C.P.Lond., Medical Officer to the Huddersfield Elementary Schools. **John Alexander Drake,** M.B.Lond., M.R.C.S.,Eng., L.R.C.P.Lond., to the Medical Staff of the Tenby Cottage Hospital. **Arthur George Haydon,** M.D.Edin., Consulting Physician to the Torrington Sanatorium, Chesham, Bucks. **Charles Christopher Heywood,** M.A., M.B.Cantab., M.R.C.P.Lond., Physician to the Manchester Children's Hospital. **Louis F. Knuthsen,** M.D., C.M.Edin., Permanent Clinical Assistant to St. John's Hospital for Diseases of the Skin, Leicester Square, W.C. **Leslie Milburn,** M.R.C.S., L.R.C.P.Lond., D.P.H., Medical Officer of Health of the Borough of Tynemouth. **James Phillips,** F.R.C.S.E., Honorary Assistant Surgeon to Bradford (Yorks) Royal Infirmary. **R. R. Pirrie,** M.B., B.S.Durh., Medical Officer to the Post Office at Ryton, including Greenside. **V. H. Rutherford,** M.B.Cantab., Medical Officer to the Electrical Light and $x$-Ray Department of St. John's Hospital for Diseases of the Skin, Leicester Square, W.C. **R. H. J. Swan,** M.S., M.B., F.R.C.S., Assistant Surgeon at the Cancer Hospital, London. **R. H. Tribe,** M.R.C.S., L.R.C.P.Lond., Anæsthetist to the Metropolitan Hospital, Kingsland Road, N.E.

### THE SCIENTIFIC PRESS, LTD., NURSING PUBLISHERS and BOOKSELLERS.

***Two Valuable Guides to those desirous of taking up Nursing as a Profession.***

### HOW TO BECOME A NURSE.

### THE NURSING PROFESSION:

**How and Where to Train.**

Edited by Sir HENRY BURDETT, K.C.B.

Cloth, 2/- net, 2/4 post free.

An invaluable Guide to would-be Nurses. It gives full details of Training Schools, Premiums, Salaries, the Hours of Duty, &c. &c.

*JUST PUBLISHED.*

### NURSING:

**Hints to Probationers on Practical Work.**

By MARY H. ANNESLEY VOYSEY.

2/- net, 2/3 post free.

A practical guide to the duties of a nurse probationer. The work will prove of the greatest assistance, and should be read by all who are contemplating nursing as a profession.

*NOW READY.*

### MEDICAL ELECTRICITY AND THE LIGHT TREATMENT.

By KATE NEALE.

Cloth, profusely Illustrated, 2/6 net, 2/9 post free.

A practical work on a subject hitherto untouched from a Nursing standpoint. The book cannot fail to be of the utmost value to all Nurses who desire to move with the times.

### SIMPLE SANITATION:

**The Practical Application of the Laws of Health to Small Dwellings.**

By M. LOANE.

With Introductory Chapter on Sanitary Legislation by Dr. A. Mearns Fraser.

Cloth, Illustrated, 1/- net, 1/1 post free.

THE BOOK FOR THE C.M.B.

### A COMPLETE HANDBOOK OF MIDWIFERY.

By J. K. WATSON, M.D. Edin.

143 Illustrations. 6/- net, 6/4 post free.

### THE NURSING OF SICK CHILDREN.

By Dr. BURNET.

Cloth, 1/- net, 1/1 post free.

A valuable treatise on the nursing of sick children. The book should prove useful to mothers as well as to nurses.

### HINTS TO NURSES ON TROPICAL FEVERS.

By S. F. POLLARD,

Sister Army Nursing Service Reserve, late Sister Guy's Hospital.

Cloth, 1/6 net, 1/8 post free.

Contents.

Hints on Going Abroad—Malaria—Dysentery—Liver Abscess—Cholera — Bubonic Plague — Beri-beri—Yellow Fever—Malta Fever—Various Fevers, &c.

*READY SHORTLY.*

### SURGICAL INSTRUMENTS AND APPLIANCES USED IN OPERATIONS.

Classified and Illustrated Lists of Instruments in General Use and for Special Operations, with Explanatory Notes.

By HAROLD BURROWS, F.R.C.S.

Limp Cloth, 1/6 net, 1/8 post free.

A work of reference of the utmost value to ***all*** nurses.

**The Scientific Press, Ltd.**

*Complete Catalogue sent post free on application.*

Publishers of Nursing Text-Books, Charts, and Case-Books of all Descriptions.

28 & 29 SOUTHAMPTON STREET, STRAND, LONDON, W.C.

